# Drug resistance in cancer immunotherapy: new strategies to improve checkpoint inhibitor therapies

**DOI:** 10.20517/cdr.2019.61

**Published:** 2019-12-19

**Authors:** Jesus Rodriguez-Pascual, Angel Ayuso-Sacido, Cristobal Belda-Iniesta

**Affiliations:** ^1^Department of Clinical Oncology, HM-CIOCC, Grupo HM Hospitales, Madrid 28050, Spain.; ^2^Fundación de Investigación HM Hospitales (FiHM), Madrid 28015, Spain.; ^3^Instituto de Salud Carlos III, Madrid 28029, Spain.

**Keywords:** Immunotherapy, checkpoint inhibitors, cytotoxic T-lymphocyte antigen 4, programmed death receptor-1, programmed death ligand-1

## Abstract

Recent advances in pharmacological immune modulation against tumor cells has dramatically changed the paradigm of cancer treatment. Checkpoint inhibitor therapy is a form of cancer immunotherapy already in clinical setting but also under active basic and clinical investigation. Nevertheless, some patients are primary unresponsive or develop ulterior resistance to these family of drugs. This review aims to update the basic molecular mechanism of resistance as well as the current strategies for checkpoint inhibitor selection in order to propose new approaches to individualize the use of these novel therapies.

## Introduction

New therapies based on modulating immune response, also known as cancer immunotherapy, has emerged as a new and promising option for tumor treatment in recent years. Relationship between immune system and tumor evolution have been described since the latest 18th century. William Coley described a group of patients suffering from locally advanced soft tissue sarcoma that presented a clinical regression after tumor infection caused by Streptococcus pyogenes (also known as Erysipellas)^[1]^. Ulterior efforts were focused on discover the relationship between immune system and tumor cells. In the 20th century, Paul Ehrlich proposed the concept of cancer immunosurveillance: emergence of cancer cells seems to be a frequent event, however, host natural immunity keeps suppressed ulterior development of tumor tissues; cancer occurs when this immune response is weakened. Linsley *et al.*^[2]^ enriched this concept focusing on T cells and its key role on immune reaction. In the last decades, molecular mechanisms of immune response were progressively clarified: T cell activation implies an antigen presentation by the major histocompatibility complex (MHC) located on the surface of antigen presenting cells (APC) to the corresponding T cell receptor (TCR) on T lymphocytes^[3]^. Costimulatory molecules CD28 and B7 interaction is required for full activation, which is regulated by inhibitor checkpoints tov avoid autoimmunity phenomenon. Agata *et al.*^[4]^ describes the cytotoxic T-lymphocyte antigen-4 (CTLA-4) receptor on activated effector T cells (Teff) and regulatory T cells (Treg). Sharma *et al.*^[5]^ demonstrate that CTLA-4 inhibits both proliferation and IL-2 secretion by T cells completing the function of CD28/B7 signal. Finally, Sanchez-Vega *et al.*^[6]^ described and cloned the programmed death 1 checkpoint receptor (PD-1), while Liu *et al.*^[7]^ and Peng *et al.*^[8]^ discovered its ligand PD-L1.

Ulterior clinical testing resulted in the approval of Anti-CTLA-4 first, and Anti-PD1 and Anti-PD-L1 monoclonal antibodies later; the last two presenting a long lasting clinical responses, with impressive plateau in survival curves in a broad spectrum of tumors, which initiate an immunotherapy era in solid and hematological tumors treatment. Despite of the initial enthusiasm, many patients do not respond to checkpoint blockade or progressed after an initial response. Molecular resistance mechanisms to checkpoint inhibitors are currently under a marked active research, however, the emerging knowledge of primary or secondary tumor resistance mechanisms does not translate into rational use of checkpoint inhibitors in clinical settings. Translation between basic research in resistance mechanisms of immunotherapy and clinical practice is key to optimize treatments, reduce cost and prolong survival in cancer patients. This review aims to update the basic molecular mechanism of resistance as well as the current strategies for checkpoint inhibitor selection in order to propose new approaches to individualize the use of these novel therapies.

## Immune response to tumor cells and the immune synapse

**Figure 1:**
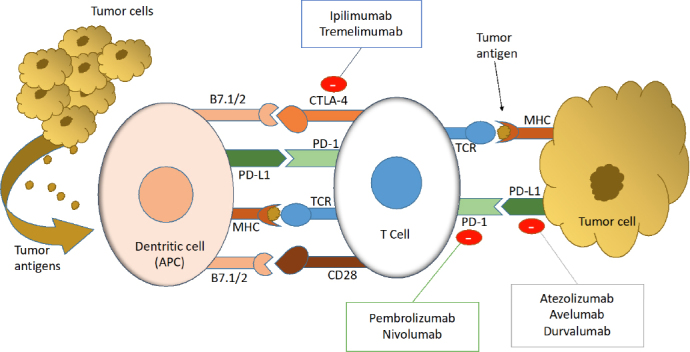
The immune synapse and response to tumor cells. APC: antigen presenting-cell; PD-L1: programmed death ligand-1; PD-1: programmed death-1; CTLA-4: cytotoxic T-lymphocyte antigen-4; TCR: T cell receptor; MHC: major histocompatibily complex; B7.1/2: ligand B7-1 and 2; CD28: cluster of differentiation-28

Immune system responds to cancer cells emergence through a coordinated response of distinct interacting cells that detect and eliminate tumor cells. The Figure 1 described the relationship among immune cells, dentritic cells and tumor cells^[1]^ (the “immune synapse”). An APC process and displays tumor antigen complexed with MHCs on their surfaces. T-cell recognize these complexes using the TCR. CTLA-4 expression and function is linked with T cell activation, and is upregulated following TCR engagement^[2]^. CTLA-4 dampens TCR signaling through competition with CD28, a costimulatory receptor for the B7 ligands B7-1 (CD80) and B7-2 (CD86)^[3]^. B7-1 and B7-2 receptors provide positive costimulatory signals through CD28, resulting in a competitive inhibition of both molecules by CTLA-4, this blockade is necessary to attenuate T cell activation.

Additionally, PD-1 is expressed upon activation of T and B lymphocytes^[4]^, and regulates immune response maintaining T cell responses within a desirable physiological range. Its ligands PD-L1 and PD-L2 are widely expressed in nonlymphoid tissues and also in tumor cells. The PD-1/PD-L1 regulatory system is induced by immune responses, forming a negative feedback loop to attenuate local T cell responses and minimizing tissue damage. PD-1 regulates T cell activation through interaction with PD-L1 and PD-L2. Inflammatory cytokines such as (interferon-gamma) INF-γ induce PD-L1 expression, and it is a key mechanism to maintain the immune response under control.

Insights into the normal immune processes and the regulation of costimulatory molecules inform our understanding of cancer immunosurveillance, mechanism of action of checkpoint inhibitors and its resistance mechanisms. Increasing the knowledge of these mechanisms is critical for the development of two future therapeutic goals: the optimization of available drugs administration in daily clinical practice and the emergence of new strategies against drug resistance.

## Mechanisms of resistance to checkpoint blockade

In primary resistance, patients do not respond to the initial treatment to checkpoint inhibitors, while in secondary resistance, patients respond initially to checkpoint inhibitors and then progressed. The underlying molecular mechanisms of primary resistance resides in the lack of response to immunotherapy, which includes adaptive immune resistance^[5]^. Adaptive resistance is a mechanism which a tumor tissue is recognized by the immune system but it protects itself by adapting to the immune response, and this effect may have a clinical translation in the form of primary resistance, mixed responses or acquired resistance. In contrast, the acquired resistance, initiates with an early response of tumor to immunotherapy that after a period of time, re-growth, relapses and progresses. Therefore, the resistant mechanisms can be divided into two major responses based on both intrinsic tumor factors or microenvironment features (called tumor cell-intrinsic and extrinsic factors). Table 1 describes the main molecular pathways implicated in mechanisms of resistance to checkpoint inhibitors^[6]^.

**Table 1:** Checkpoint inhibitors, current indications and associated biomarker

MoAb	Target	FDA/EMEA Approval	Biomarker of response/resistance
Ipilimumab	CTLA-4	Melanoma	No
		RCC (^*^)	No
Tremelimumab	CTLA-4	No	No
Pembrolizumab	PD-1	NSCLC 1st line	TPS PD-L1 expression ≥ 50%^[62]^
		NSCLC after 1st line	TPS PD-L1 expression > 1%^[62]^
		H&NSCC	TPS PD-L1 expression ≥ 50%^[62]^
		UC	CPS ≥ 10^[62]^
		cHL	Not required
Nivolumab	PD-1	Melanoma (^&^)	No
		NSCLC, H&NSCC, UC, cHL	Not required
Atezolizumab	PD-L1	UC 1st line (^$^)	≥ 5% PD-L1 expression^[63]^
		UC after 1st line, NSCLC	Not required
Avelumab	PD-L1	MCC	Not required
Durvalumab	PD-L1	NSCLC (^ç^)	≥ 1% PD-L1 expression

MoAB: monoclonal antibody; FDA: food and drug administration; EMEA: European Medicines Agency; CTLA-4: cytotoxic T-lymphocyte antigen-4; PD-1: programmed dead cell receptor-1; PD-L1: programmed dead ligand-1; RCC: renal cell carcinoma; NSCLC: non small-cell lung cancer; H&NSCC: head and neck squamous cell carcinoma; UC: urothelial carcinoma; cHL: classic hodgkin lymphoma; MCC: merkel cell carcinoma; TPS: tumor proportion score; CPS: combined proportion score; *: In combination with Nivolumab; &: In combination with Ipilimumab; ^$^: If no progression disease after chemoradiotherapy in locally-advanced tumors

### Primary resistance mechanism

#### Tumor-cell mechanism

Primary Tumor-cell resistance mechanisms suppose a wide variety of intrinsic molecular cancer-cell alterations that primary block the immune system action in response to checkpoint inhibitors.

#### RTK/RAS pathway

The mitogen-activated protein kinase (MAPK) pathway activation presents inhibitory effects on T cell recruitment and function. In this sense, BRAF inhibition in combination with adoptive T cell transfer is more effective at inducing long-term clinical regression of BRAF-mutant tumor in BRAF-mutated human melanoma tumor-cell models, providing a strong rationale for the potential application of combining BRAF inhibitors and immunotherapy^[7]^.

#### PI3K/PTEN/mTOR

The importance of PTEN status in tumor cells was explored in preclinical models of melanoma cells^[8]^, also confirming a strong correlation between tumor PTEN loss and T cell infiltration at tumor sites and supporting the rationale for further evaluate combinatorial strategies targeting PI3K-AKT to increase immune response to checkpoint inhibitors.

#### WNT pathway

Some studies support the stabilization of b-catenin resulting in constitutive WNT signaling activation as a key mechanism in melanoma models, resulting in primary resistance of checkpoint inhibitors^[9]^. WNT signaling is frequently dysregulated in melanoma. b-catenin levels control tumor differentiation and regulate both MAPK and PI3K signaling. A correlation between activation of the WNT/b-catenin signaling pathway and the absence of a T cell gene expression signature would result in poor clinical responses. However, relationship between immune expression and WNT signaling alterations still remains unclear.

#### INF-γ pathway

INF-γ pathway plays a pivotal role in molecular resistance mechanisms to immune checkpoint inhibitors. Anti-CTLA blockade could be overcome in patients treated with melanoma in base to primary or secondary alterations in INF-γ pathway genes^[10]^, at least in mouse models. In fact, mice bearing melanoma tumors with knockdown of INF-γ receptor 1 have impaired tumor rejection upon anti-CTLA-4 treatment, illustrating that loss of the INF-γ signaling pathway is associated with primary resistance to checkpoint blockade. In this context, recent studies involving the INF-g-JAK1/JAK2-STAT1/STAT2/STAT3-IRF1 axis describes an inducible expression of PD-L1 (with IRF1 binding to its promoter) and PD-L1 (with STAT3 bind to its promoter) confirming the primary role in immune synapse regulation.

#### Cyclin and cell cycle pathway

Cyclin-dependent kinase 5 (Cdk5) function^[11]^ allows some tumor models as medulloblastoma (MB) to evade immune elimination via INF-g-induced PD-L1 up-regulation. Thus, MB PD-L1 expression results in potent CD4(+) T cell-mediated tumor rejection. In this tumor model, loss of Cdk5 results in persistent expression of IRF2 and IRF2BP2, which likely leads to reduced PD-L1 expression on tumors thereby promoting anti-tumor immunity.

#### Epigenetic control: DNA methylation

Epigenetic modification of DNA may play an important role in primary resistance to immunotherapy. In preclinical models, deacetylase inhibitors increase MHC and tumor-associated antigen expression, resulting in a decrease of competing endogenous lymphocytes and a proliferative advantage for the adoptively transferred cells^[12]^. Epigenetic modulation can induce the expression of a major T cell co-stimulatory molecule on cancer cells, which in turn overcome immune tolerance, and induce an efficient anti-tumor CTL response.

#### PD-L1 expression and molecular control

A key mechanism in tumor resistance to immunotherapy implies a constitutively expression of cell surface ligands that may inhibit antitumor T cell responses. This mechanism involves, but not only, proteins related to immune synapse discussed above. Specifically, increased PD-L1 expression may inhibit antitumor T cell responses, and it is a critical aspect that explains the main mechanism of checkpoint inhibitors. An increase of PD-1 constitutive expression is described on classical Hodgkin lymphoma (cHL) and mediastinal large B-cell lymphoma cell lines^[13]^, identifying PD-L1 and PD-L2 as key targets at the 9p24.1 amplification region, that includes the Janus kinase 2 (JAK2) locus. 9p24.1 amplification is a disease-specific structural alteration that increases both the gene dosage of PD-1 ligands and their induction by JAK2, defining the PD-1 pathway and JAK2 as complementary therapeutic targets. In fact, in non-small cell lung cancer cell lines^[14]^, oncogenic activation of the AKT-mTOR pathway promotes immune escape by driving expression of PD-L1. Additionally, EGFR-driven lung tumors inhibit antitumor immunity by activating PD-1/PD-L1 pathway to suppress T cell function and increase levels of proinflammatory cytokines^[15]^.

MYC pathway also regulates the expression of PD-L1 and CD47^[16]^, an innate immune regulator. Thus, MYC inactivation in mouse tumors down-regulated CD-47 and PD-L1 expression and enhanced the antitumor immune response; in contrast, when MYC was inactivated in tumors with enforced expression of CD47 or PD-L1, the immune response was suppressed, and tumors continued to grow.

#### Microenvironment mechanisms

Other components besides tumor cells contribute to primary or secondary resistance to checkpoint inhibitors, which include Tregs, myeloid derived suppressor cells (MDSCs), M2 macrophages, fibroblast, and other stromal cells.

Regulatory T cells are critical lymphocytes maintaining self-tolerance. Tregs can be identified by expression of FoxP3 transcription factor^[17]^, and are known to suppress Teff responses by secretion of inhibitory cytokines such as IL-10 and TGF-beta or direct cell contact^[18]^. Many human tumors are infiltrated by Tregs^[19]^, and its depletion from the tumor microenvironment can enhance or restore anti-tumor immunity. Clinical responses to checkpoint inhibitors have been associated with augmentation of preexisting immune responses, however, many other tumors have a non-inflammed microenvironment.

Preliminary studies highlight the MDSCs as major regulators of immune responses in cancer. MDSCs were characterized by the expression of CD11b and Gr-1 markers^[20]^ and they are implicated in promoting tumor cell invasion and metastasis^[21]^. By other hand, the presence of MDSCs correlates with reduced survival in colorectal and breast cancer patients^[22]^. The presence of MDSCs in the tumor microenvironment also correlates with decreasing efficacy of immunotherapies, including checkpoint inhibitors^[23]^.

Tumor-associated macrophages (TAMs), including M1 and M2 macrophages are another subgroup of white cells that seem to influence responses to immune therapies. M1 macrophages are involved in promoting anti-tumor immunity, while M2 macrophages possess pro-tumorigenic properties^[24,25]^. Some reports suggest that macrophages can suppress T cell responses through PD-L1^[26]^.

How the genomic landscape of a tumor is shaped by anti-tumor immunity has been explored using large-scale genomic data sets of tumor biopsies across different tumor types^[27]^, defining some recurrently mutated genes that showed positive association with antitumor activity, including beta-2-microglobulin (β2M), HLA-A, -B and -C and Caspase 8. A resulting T cell gene expression profile can predict response to checkpoint inhibitors in a broad spectrum of tumor types.

### Secondary resistance mechanisms

The potential mechanism of resistance after primary response to checkpoint blockade include development of escape mutations variants in cancer cells, lack of T cell recognition by downregulation of tumor antigen presentation and loss of T cell function^[5]^. Mutations, genetic deletions or epigenetic changes could lead to loss of expression of mutational neoantigens presented by MHC molecules, resulting in acquired resistance to checkpoint inhibitors.

Inhibitory immune checkpoints that are often expressed in the tumor microenvironment include Lag-3, Tim-3 and TIGIT: these receptors, although they belong to the same class or receptor as PD-1 and CTLA-4 exhibit unique functions, especially at tissue sites where they regulate distinct aspects of immunity and are under active investigation^[28]^.

In a recent study, acquired resistance to PD-1 blockade immunotherapy in patients with melanoma was associated with defects in the pathway involved in interferon-receptor signaling JAK1or JAK2, concurrent with deletion of the wild-type allele^[29]^. A truncating mutation in the gene encoding the β2M was also identified. This mutation led to loss of surface expression of major MHC class I interfering the antigen presentation.

Another evidence of loss of antigen presenting machinery leading to acquired resistance to cancer immunotherapy is provided identifying a polyclonal CD8+ T cell response against KRAS G12D in tumor-infiltrating lymphocytes obtained from a patient with metastatic colorectal cancer^[30]^. Objective regression was observed of lung metastasis after the infusion of HLA-C*8:02-restricted tumor infiltrating lymphocytes that were composed of four different T cell clonotypes that specifically targeted KRAS G12D. However, one lung lesion progressed on evaluation 9 months after therapy: the lesion was resected and found to have lost the chromosome 6 haplotype encoding the HLA-C*8:02 class I MHC molecule. The loss of expression of this molecule provided new evidence of secondary immune evasion.

## Checkpoint inhibitors and current predictive biomarkers

### Checkpoint inhibitors in clinical practice

Clinical development of checkpoint inhibitors has been complemented in some cases with the inclusion in clinical practice of predictive biomarkers. Table 1 correlates drug, tumor indications and approved biomarker in every histological type.

#### Ipilimumab

This is a fully human IgG1 monoclonal antibody that blocks CTLA-4 increasing the number of reactive T-effector cells which mobilize a direct T-cell immune response against tumor cells^[31,32]^. Reduction of Treg function mediated by CTLA-4 blockade and Treg depletion (increasing the intratumoral Teff/Treg ratio) can also reduce Treg function contributing to an antitumor effect^[33]^. As monotherapy, Ipilimumab is indicated for the treatment of advanced melanoma^[34]^ and, in combination with Nivolumab, it is indicated for the treatment of advanced melanoma and renal cell carcinoma (RCC)^[35]^.

Ipilimumab was the first checkpoint inhibitor that showed activity in solid tumors, demonstrating a clear benefit in overall survival (OS) in two phase 3 studies involving patients with metastatic melanoma^[36,37]^. It is also the first monoclonal antibody that showed increased activity in combination with other checkpoint inhibitor, Nivolumab, a monoclonal antibody which binds to the PD-1 receptor^[38]^.

There is no required biomarker needed in the treatment of melanoma in monotherapy. Relative to Nivolumab combination in the treatment of RCC, low tumor PD-L1 expression predicts progression-free survival (PFS) and OS, but it is not a condition to administrate with Ipilimumab^[35]^.

#### Tremelimumab

This is a fully human anti-CTLA4 monoclonal antibody under current investigation. The antitumor activity in melanoma patients showed contradictory results^[39,40]^. Ongoing studies in pancreatic cancer^[41]^, hepatocellular carcinoma^[42]^, NCSCL^[43]^ or malignant mesothelioma^[44,45]^ will define its role in the standard of care in the future clinical practice. Currently, the combination of Tremelimumab and Durvalumab, an antibody against the PD-L1 receptor (see above) seems to be an active combination in head and neck carcinomas and NSCLC (see above).

#### Pembrolizumab

Pembrolizumab is a humanized monoclonal antibody (IgG4/kappa isotype) which binds to de PD-1 receptor and blocks its interactions with ligands PD-L1 and PD-L2, potentiating anti-tumor responses. As monotherapy, Pembrolizumab is indicated for the adjuvant treatment of stage III melanoma after complete resection and, in the metastatic setting, for advanced melanoma, non-small cell lung carcinoma (NSCLC), relapse or refractory cHL, platinum-resistant urothelial carcinoma and head and neck squamous cell carcinoma (HNSCC)^[46]^.

In advanced NSCLC, Pembrolizumab is indicated in the first-line treatment if tumor expression of PD-L1 presents ≥ 50% of tumor proportion score (TPS) and, after chemotherapy (at least one prior chemotherapy regimen), if tumor expression of PD-L1 presents ≥ 1% of TPS. In HNSCC, it is indicated only in patients whose tumors progressing on or after platinum containing chemotherapy and express PD-L1 with ≥ 50% TPS. In advanced urothelial carcinoma in patients not eligible for cisplatin-containing chemotherapy, Pembrolizumab is indicated if tumor express PD-L1 with a combined positive score (CPS) ≥ 10. No biomarkers of response are needed in melanoma or Hodgkin lymphoma.

#### Nivolumab

Nivolumab is a human immunoglobulin G4 monoclonal antibody which binds to the PD-1 receptor and blocks its interaction with PD-L1 and PD-L2. Combined Nivolumab and Ipilimumab (anti-CTLA-4) mediated inhibition results in improved anti-tumor responses in metastatic melanoma. Thus, in monotherapy it is indicated in melanoma patients both in the adjuvant and in the metastatic setting. It is also approved in combination with Ipilimumab in advanced melanoma. In NSCLC, it is used in locally advanced or metastatic patients after prior chemotherapy. In RCC as monotherapy or in combination with Ipilimumab, it is indicated for the first-line treatment of adults with intermediate/poor-risk tumors. Finally, Nivolumab is an active treatment and it is indicated in relapsed or refractory cHL, recurrent or metastatic platinum-resistant SCCHN and after platinum-based therapy of urothelial carcinoma^[35]^.

Relative to Nivolumab monotherapy in melanoma patients, an increase in PFS and OS for the combination of Nivolumab and Ipilimumab is established only in patients with low tumor PD-L1 expression.

#### Atezolizumab

Atezolizumab is a monoclonal antibody that directly binds to PD-L1 and provides a dual blockade of the PD-L1 and B7.1 receptors, releasing PD-L1/PD-1 mediated inhibition of the immune response, including reactivating the antitumor immune response. Atezolizumab spares the PD-L2/PD-1 interaction allowing PD-L2/PD-1 mediated inhibitory signals to persist. Atezolizumab is indicated for the treatment of urothelial carcinoma after a platinum-containing regimen, and in NSCLC after prior chemotherapy^[47]^.

Patients with previously untreated urothelial carcinoma should be selected for treatment based on the tumor expression of PD-L1: if patients are considered ineligible for platinum-containing regimen, PD-L1 expression 5% permit Atezolizumab administration. After first line chemotherapy regimen, urothelial carcinoma and NSCLC patients can receive Atezolizumab irrespectively of PD-L1 expression.

#### Avelumab

Avelumab is a human monoclonal IgG1 antibody directed against the PD-L1 receptor. It is indicated as monotherapy for the treatment of adult patients with metastatic Merkel cell carcinoma (MCC)^[48]^, a rare, aggressive skin cancer. In the pivotal phase 2 trial^[49]^ patient selection was not based on PD-L1 expression. A recent phase III trial^[50]^ demonstrated a PFS combining Avelumab plus Axitinib among patients with PD-L1 positive advanced renal-cell carcinoma compared with the first line standard-of-care Sunitinib. Avelumab is under current investigation in urothelial carcinoma^[51]^, breast cancer^[52]^, NSCLC and other solid tumors.

There is no described biomarker of response or primary resistance in MCC patients currently treated with Avelumab.

#### Durvalumab

Durvalumab is a human monoclonal IgG1 antibody directed against the PD-L1 receptor. It is indicated for the treatment of locally advanced, unresectable NSCLC whose disease has not progressed following platinum-based chemoradiation therapy^[53,54]^. In clinical trials, Durvalumab presented antitumor activity against NSCLC alone or in combination with Tremelimumab^[55]^. This combination seems to be active against recurrent or metastatic HNSCC in low or no PD-L1 tumor cell expression, suggesting an increasing in tumor or microenvironment immunogenicity due to combination treatment^[56]^. Durvalumab is also active against urothelial carcinoma^[57,58]^. The combination with a poly (ADP-Ribose) polymerase inhibitor Olaparib and a vascular endothelial growth factor receptor 1-3 inhibitor Cediranib presented antitumoral activity in preliminary studies^[59]^.

In NSCLC patients, Durvalumab is indicated in patients whose tumors express PD-L1 on ³ 1% of total tumor cells.

### Predictive biomarkers in current clinical practice

Table 2 shows the response checkpoint inhibitor biomarkers approved in daily clinical practice using for solid tumor treatment.

**Table 2:** Biomarkers for checkpoint inhibitors

Biomarker	Tumor Type	Definition
Tumor Proportion Score	NSCLC	Percentage of tumor cells expressing the protein PD-L1 relative to the total number of tumor cells
Combined Proportion Score	UC H&NSCC	Percentage of PD-L1 expression in tumor and infiltrating immune cells relative to the total number of tumor cells
MSI-H	Tumor Agnostic	Microsatellite Instability

PD-L1: programmed dead ligand-1; NSCLC: non small-cell lung cancer; H&NSCC: head and neck squamous cell carcinoma; UC: urothelial carcinoma

#### PD-L1 expression and predictive scores

Knowledge of the role played by each receptor modulating the immune synapse has allowed the development of some useful response biomarkers. The most important biomarker in routine clinical practice is determination of the expression of PD-L1 on tumor cells surface. Thus, the first clinical studies with Nivolumab in a wide spectrum of tumors^[60]^ found that response to checkpoint inhibitors were restricted to patients who expressed PD-L1, while patients without PD-L1 tumor expression were frequently unresponsive.

On the other hand, a preliminary study of Atezolizumab followed an adaptive design to allow some PD-L1-positive enriched cohorts^[61]^. Herbst *et al.*^[61]^ establish IHQ expression levels in relation to the percentage of tumor cells expressing the protein, as 0 (< 1%), 1 (1%-5%), 2 (> 5%-10%) or 3 (> 10%). Again, a positive correlation was found between patients with tumors with positive PD-L1 expression and the inhibitory checkpoint response, in this case against the direct blockade of PD-L1. The key concept of a positive, preferred association between PD-L1 expression and tumor response, independent of PD-L1 expression has emerged as a key concept in oncology.

Thus, in advanced melanoma, there is a correlation between presence of intratumoral CD8+ T cells and response to Pembrolizumab^[62]^, confirming the need for a preliminary presence of peritumoral CD8+ T-cells together with the inhibitory response of the PD-1/PD-L1 axis as requirements for a potent antitumor response. Likewise, the presence of CD8- T cells in subsequent biopsies after progression in these patients tends to decrease, reflecting an important mechanism of secondary resistance.

In fact, response to checkpoint inhibitors based on expression of PD-L1 presents a continuous gradation: there is a clear positive correlation between protein expression and tumor response, and this correlation is especially intense in patients with high PD-L1 expression of PD-L1. This phenomenon was described in studies that led to the approval of Pembrolizumab in NSCLC. In the KEYNOTE-001 study, Garon *et al.*^[63]^ assessed PD-L1 expression in solid tumor samples in training group and a validation group, defining the percentage of membranous tumor PD-L1 expression as a proportion score. Thus, patients who presented a PD-L1 of at least 50% presented higher response rates and longer OS. These responses remain prolonged over time^[64]^. Currently, the NCCN^[65]^ NSCLC Panel recommends IHC testing for PD-L1 expression before first-line treatment in patients with metastatic NSCLC, however, there are no unified criteria for the administration of the entire set of all the different checkpoint inhibitors in relation to expression levels of PD-L1. In spite of this, assessing PD-L1 expression in locally-advanced disease after chemo-radiotherapy and in first line after progression to a systemic treatment seems to be a reasonable strategy.

Solid bladder urothelial carcinoma is another type of tumor in which good responses have been verified with checkpoint inhibitors. The active drugs administer are Prembrolizumab^[66]^, Atezolizumab^[67]^, Nivolumab^[68]^, Avelumab^[69]^ or Durvalumab^[58]^. However, the value of PD-L1 expression as a predictive response factor showed disparate results. A specific response score (PD-L1 or CPS) was determined in urothelial carcinoma patients treated with Pembrolizumab: his was defined as the percentage of PD-L1 expression in tumor and infiltrating immune cells relative to the total number of tumor cells, with positive markers scoring higher than 10%. Unexpectedly, the benefit of the monoclonal antibody appeared to be independent of PD-L1 expression on tumor and infiltrating cells^[66]^.

Finally, checkpoint inhibitors are active drugs against HNSCC. Both Nivolumab^[70]^ and Pembrolizumab^[71,72]^ showed antitumor activity in recent clinical trials. In the same way, the latest studies with Pembrolizumab include determination of PD-L1^[73]^. In a second interim analysis of this Phase III trial comparing single-agent Pembrolizumab versus platinum-based chemotherapy or the combination, patients with CPS > 20 showed a better OS.

#### MSI-H status

Microsatellite Instability is a biological condition that results from inactivation of the DNA repair system known as Mismatch Repair (deficient Mismatch Repair or dMMR). It is characterized by a large increase in point mutations, which are detected by a marked increase in sequence instability in chromosomal regions called microsatellites (High Microsatellite Instability or MSI-H). Inactivation of MLH1, MSH2, MSH6 or PMS2 Mismatch Repair protein can occur sporadically (usually by inactivated expression caused by promoter methylation) or less frequently as a germline mutation (Lynch Syndrome)^[74,75]^.

MSI-H condition presents a high predictive value in terms of response to checkpoint inhibitor therapies^[76]^. MSI-H tumors shows a marked clinical response in comparison with MMR proficient tumors. Currently, the US Food and Drug Administration has approved Pembrolizumab for the treatment of unresectable or advanced solid tumors after standard treatment (especially in colorectal cancer after oxaliplatin, irinotecan and fluoropyrimidine progression, or in first line advanced NSCLC). It is important to note that antitumor activity is not restricted to a specific tumor type (“site agnostic”). Therefore, now in 2019, the possibility of using Nivolumab and the combination of Nivolumab-Ipilimumab has been opened. In fact, MSI-H is predictive of Lynch Syndrome across a much broader tumor spectrum than currently appreciated, such urothelial, melanoma, gastric or gem cell tumors. However, use of checkpoint inhibitors in this scenario is currently under active investigation.

## New approaches to optimize the activity of checkpoint inhibitors: rational development of clinical trials

**Figure 2:**
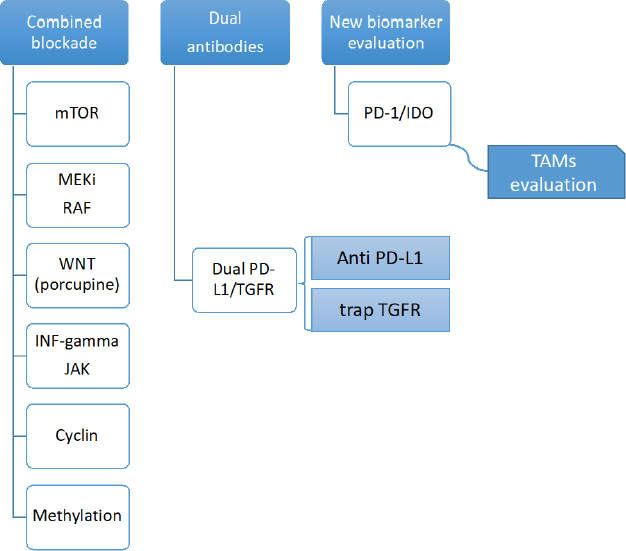
Strategies to optimize the antitumor activity of checkpoint inhibitors

The most important strategies to optimize the antitumoral activity of checkpoint inhibitors in clinical research are described in Figure 2. Notably, a first strategy focuses on combining anti PD-1 or PD-L1 monoclonal antibodies with a second drug that blocks other molecular signaling pathways, especially those related to mechanisms of resistance to checkpoint inhibitors. An alternative approach involves the design of monoclonal antibodies with dual activity, which can modulate the action of PD-1 or PD-L1 blockade. Bispecific antibodies can act in base to a synergistic effect by simultaneously modulating the activity of two immunoregulating targets. Finally, development of new response biomarkers, such as the infiltration of TAMs in tumor specimens, can optimize the rational use of checkpoint inhibitors in clinical setting. The following table summarizes several approaches in current clinical and translational research to optimize the use of checkpoint inhibitors.

The following table lists some ongoing clinical studies that show different strategies for the improvement of immunotherapy based on checkpoint inhibitors.

## Conclusion

Development of checkpoint blockade-based therapy is an important medical issue in the treatment of some aggressive tumors, such as melanoma, NSCLC or urothelial carcinoma. Knowledge of the mechanisms of tumor response and resistance are currently under active investigation. However, this is not reflected in our clinical practice: the percentage of PD-L1 expression, TPS or the CPS are only partially informative biomarkers to evaluate primary resistance.

By other hand, other studies focused on clinical factors, such as laboratory data, specific site of metastasis or clinical comorbidities could be added to a comprehensive evaluation of resistance of checkpoint inhibitors^[77,78]^.

To date, there are no validated studies in clinical practice to test for secondary resistance to checkpoint inhibitors, and in primary resistance (based on the existence of MSI or based on the PD-L1 expression) these appear to be insufficient. A concerted effort is, therefore, required to understand all these biological mechanisms and to apply them in routine clinical practice.

## Declarations

### Copyright

© The Author(s) 2019.
